# Microwave Backscatter-Based Wireless Temperature Sensor Fabricated by an Alumina-Backed Au Slot Radiation Patch

**DOI:** 10.3390/s18010242

**Published:** 2018-01-16

**Authors:** Fei Lu, Haixing Wang, Yanjie Guo, Qiulin Tan, Wendong Zhang, Jijun Xiong

**Affiliations:** Science and Technology on Electronic Test & Measurement Laboratory, North University of China, Taiyuan 030051, China; lufei_55@163.com (F.L.); whx19920414@163.com (H.W.); 18235140097@163.com (Y.G.); wdzhang@nuc.edu.cn (W.Z.); xiongjijun@nuc.edu.cn (J.X.)

**Keywords:** wireless temperature sensor, microwave backscatter, slot radiation patch, RFID tags, resonant frequency

## Abstract

A wireless and passive temperature sensor operating up to 800 °C is proposed. The sensor is based on microwave backscatter RFID (radio frequency identification) technology. A thin-film planar structure and simple working principle make the sensor easy to operate under high temperature. In this paper, the proposed high temperature sensor was designed, fabricated, and characterized. Here the 99% alumina ceramic with a dimension of 40 mm × 40 mm × 1 mm was prepared in micromechanics for fabrication of the sensor substrate. The metallization of the Au slot patch was realized in magnetron sputtering with a slot width of 2 mm and a slot length of 32 mm. The measured resonant frequency of the sensor at 25 °C is 2.31 GHz. It was concluded that the resonant frequency decreases with the increase in the temperature in range of 25–800 °C. It was shown that the average sensor sensitivity is 101.94 kHz/°C.

## 1. Introduction

The development of sensors for high-temperature applications is now on its way. There are various fields that are in urgent need of sensing devices tolerant to high temperatures. For instance, engine turbine blades operate at a high speed of rotation when the aircraft is in operation, thus causing a high temperature on the surface of the blades and deformation of the alloyed blades due to the interaction between rotated blades and the airstream. It is essential to monitor the changeable parameters relevant to rotated blades in situ so that the operating life of the engines can be extended [1,2,3,4].

For sensing devices applied in harsh environments as mentioned above, conventional sensors are often wire-connected, such as the thermocouple sensor proposed in [5]. A thin-film structure can be directly printed on the object of interest and can be integrated with the measured turbine blades. Thermocouple sensors have a low profile and a high working temperature, but this thin-film sensor cannot work for a long period of time at a high speed of rotation because its wires are connected to a data acquisition unit; otherwise, the rotated object would be negatively influenced [6,7]. For instance, the airflow used for cooling the blades would be perturbed and the turbine performance would be negatively influenced [1]. Such wireless and passive sensors are required for contactless measurement under harsh environments. Tan et al. proposed an LC resonant sensor in [8] that can work up to 700 °C, realizing a wireless measurement in temperature. However, the measured signal was rapidly reduced due to its relatively lower Q factor, causing a short sensing distance. In addition, the lumped circuit model generally works at low frequency for its large size under high frequency up to a GHz magnitude. Recently, microwave backscatter technology was developed to overcome this weakness. Cheng et al. [9] fabricated a wireless temperature sensor using an integrated cylindrical resonator/antenna structure, realizing wide-range temperature measurement from 25 to 1300 °C. However, the metal-closed cylindrical structure made the sensor high-profile. Tan et al. [10] proposed a thin-film antenna-resonator integrated wireless temperature sensor based on a low-temperature co-fired ceramic that can operate up to 400 °C with a sensing distance of 30 mm. It can work in high frequency of 2.28 GHz with a measurement sensitivity of 0.24 MHz/°C.

Recently developed RFID (radio frequency identification) is a kind of wireless sensing technology that can automatically recognize and track the informative electronic tags connected to objects. For this technology, the electromagnetic microwave is viewed as the medium that carries a great deal of information about the objects. Compared with traditional bar codes, one of the biggest advantages is that the RFID tags can be embedded in objects that are being tracked, while not necessarily distributed on the surface of the objects. There have been many RFID-based sensors that can sense variable environmental parameters [11,12], including pipeline integrity and humidity, even on individual cell tracking and monitoring [13].

In this paper, we propose a wireless and passive temperature sensor based on the microwave backscatter RFID tags that can work up to 800 °C with an average sensitivity of 101.94 kHz/°C. The sensor was a thin film structure with Au metallization on the alumina substrate. It was fabricated in micromechanics and magnetron sputtering technology. This may have applications in practical engineering fields, such as aerospace, communication and transportation, and manufacturing, because of the possibility of realizing thin film structures with 3D printing technology. These thin films might be able to be printed on the measured device without negatively influencing them, even for mobile objects, whether the object of interest is metallic, flat, or cambered.

## 2. Design and Fabrication of the Sensor

**Figure 1:**
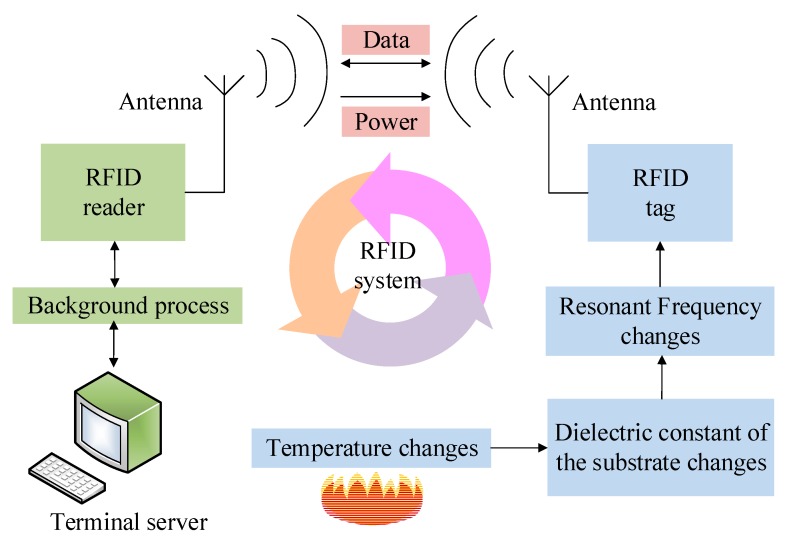
The flow chart of the wireless RFID (radio frequency identification) sensor operation.

Figure 1 illustrates the operation principle of the designed sensor, in which an RFID system is composed of an RFID reader accompanied by an antenna, a terminal server that can achieve the function of data background process and display, and an RFID tag along with an antenna. Both of the antennas take on the functions of the emission and reception of the electromagnetic wave signals that carry power and data related to the object. The working principle of the RFID temperature sensing system designed in this paper is apparent in Figure 1. When there is increase in the environmental temperature, the dielectric constant of the sensor substrate increases, causing an increase in the resonant frequency of the RFID tag-based sensor. The frequency variation can be wirelessly detected and sent to the RFID reader (a vector network analyzer). Here the temperature-dependent data is received by a readout antenna (a commonly used microstrip antenna) and displayed on the terminal server. Hence, the temperature is wirelessly measured.

**Figure 2:**
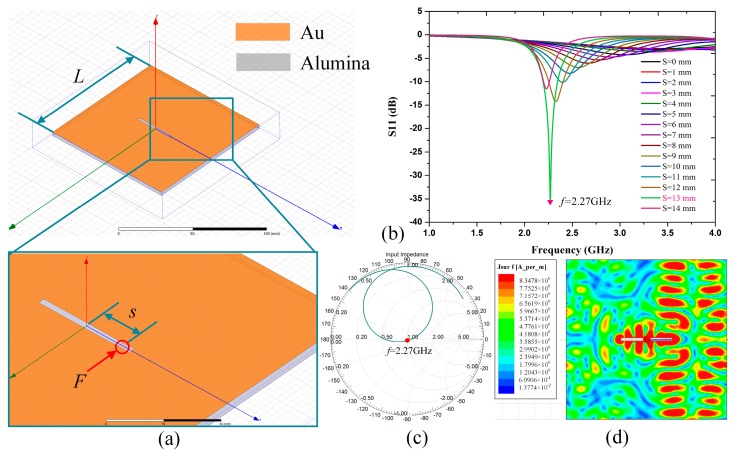
HFSS design of the sensor. (**a**) 3D view in HFSS. (**b**) The simulated frequency versus S(1,1) with the feeding point’s location *S* = 0, 1, 2,…14 mm, respectively. (**c**) The Smith Chart of impedance with reference to 50 Ω feeding port and (**d**) surface current distribution when the sensor is fed with *S* = 13 mm at resonant frequency of 2.27 GHz.

The temperature sensor is designed as a slot radiation patch with an alumina substrate backing, which is based on microwave backscatter. Figure 2a shows a slot radiator with a resonant frequency of *c*/*λ* (*c* denotes the speed of light in vacuum, and *λ* denotes the wavelength). This radiator is composed of a planar metal patch with a punched *λ*/2 slot and an alumina substrate. *F* denotes the feeding point, and the feeding port has a characteristic impedance of 50 Ω. When the slot patch is fed at the feeding point, there will be electromagnetic wave radiated by the patch according to microwave backscatter theory. It can be seen from Figure 2b that, when the slot of the patch is fed with the feeding point’s location *S* = 13 mm, the S(1,1) amplitude of the interrogation antenna reaches maximum with the sensor resonant frequency of 2.27 GHz. Figure 2c,d shows the Smith Chart and the surface current distribution of the excited patch.

**Figure 3:**
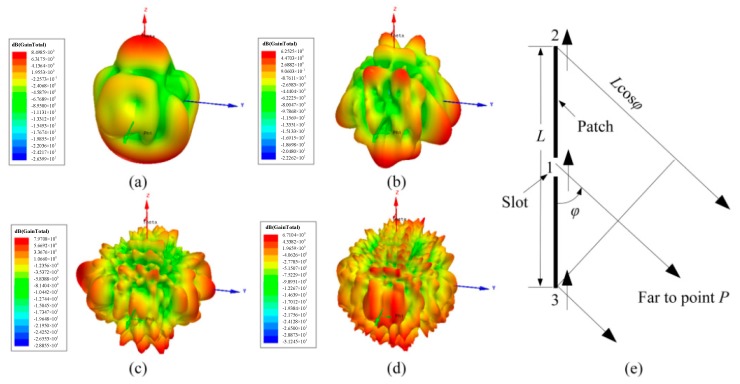
The simulated 3D gain total of the slot patch with a slot width of 2 mm and a length of 32 mm, where *L* = (**a**) 40 mm, (**b**) 100 mm, (**c**) 200 mm, and (**d**) 300 mm, respectively. (**e**) The cross-sectional view of the finite length patch.

The simulated 3D gains of the slot patch with patch length *L* = 40 mm, 100 mm, 200 mm, and 300 mm are shown in Figure 3a–d. It can be seen that the radiation gains are distributed along *x*, *y*, and *z*. They are not symmetrically distributed at the two sides of the *x*–*y* plane due to the existence of the alumina substrate. When *L* is 40 mm, the lobe pattern around the patch is corrugated with a larger surface bulge. With the increase in *L*, the corrugation increases and the fluctuation degree weakens. Hence, if the length of the patch is much longer, the lobe pattern will be close to a sphere, which can be concluded by comparison with Figure 3a–d. Staff of the Radio Research Laboratory Harvard University [14] used the Andrew Alford method to describe a method of determining the orientation of the maximum and minimum radiation in the lobe pattern. It was assumed that the far field of the radiation patch is generated by three point sources, 1, 2, and 3, as shown in Figure 3e. Point 1 is located in the slot with the electric field intensity of *sinωt*. Points 2 and 3 are located at the edge of the patch with the electric field intensity of *ksin*(*ωt − δ*). Here *k* << 1, and *δ* is the phase difference between Sources 1 and both 2 and 3. Hence, the relative electric field intensity at Point *P* can be expressed as

(1) E=sinωt+ksin(ωt−δ−ε)+ksin(ωt−δ+ε)

where *ε* = *(π/λ)L*cos*φ*. By dint of trigonometric function expansion, Equation (1) can be rewritten as

(2) E=(1+2kcosδcosε)sinωt−(2ksinδcosε)cosωt.

The module of vector *E* can be calculated as

(3) $$\left|E\right|=\sqrt{{\left(1+2k\cos\delta \cos\varepsilon \right)}^{2}+{\left(2k\sin\delta \cos\varepsilon \right)}^{2}}.$$

Ignoring the items that contain *k*^2^, Equation (3) can be simplified as

(4) $$\left|E\right|=\sqrt{1+4k\cos\delta \cos\varepsilon }.$$

It can be concluded that |*E*| is the function of *ε*, and the maximum and minimum values are calculated when *ε* = *nπ*, i.e.,

(5) $$\varepsilon =\frac{\pi }{\lambda }L\cos\varphi =n\pi$$

where *n* denotes an integer and Equation (5) can be calculated as

(6) $$\cos\varphi =\frac{n\lambda }{L},\phi =\arccos\frac{n\lambda }{L}.$$

Hence, the orientations *φ* of the maximum and minimum radiation in the lobe pattern are independent of *k* and *δ*.

For the fabrication of the sensor, the substrate was prepared via micromechanics technology. Ninety-nine percent alumina ceramic was chosen for its high-temperature resistance, which can survive up to 1500 °C. The properties of the 99% alumina ceramic, which was provided by Bolandi Ceramic Processing Ltd. (Taiyuan, China), are shown in Table 1, For our sensor designed via calculation and HFSS 13.0 software, a rectangular sheet with a dimension of 40 mm × 40 mm × 1 mm was prepared. Then, magnetron sputtering technology was used for the metallization of the microwave slot radiation patch. Here, an Au target was utilized for its high-temperature resistance, as Au can tolerate up to 1000 °C. It should be noted that, to eliminate the influence of skin effects, the thickness of the printed metal should be at least ten times the skin depth [15]. The skin depth of specific metallic conductor is related to resonant frequency *f*_0_ and can be expressed as

(7) $$\delta =\frac{2}{\sqrt{2\pi \mu_{0}\sigma f_{0}}}$$

where *δ* denotes the skin depth of specific metallic conductor, *μ*_0_ represents the relative permeability of the vacuum, and σ is the conductivity. The Au used for the fabrication of the sensor was 4.52 × 10^7^ S/m. The skin depth can be calculated to be 0.70 μm, corresponding to a frequency of 2.31 GHz. However, the Au metallization pattern cannot be sputtered for 7.0 μm due to the low speed of the magnetron sputtering apparatus; otherwise, a couple of hours would be needed for fabrication of the slot patch. Therefore, the metallization thickness for Au was chosen to be 800 nm, which was higher than the skin depth of 0.70 μm in consideration of its high-frequency skin effect.

**Figure 4:**
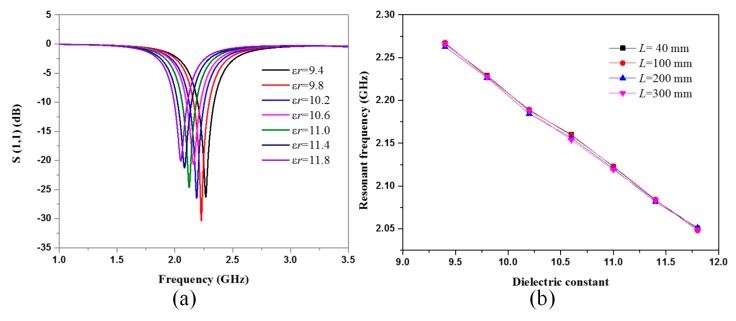
The frequency sweep simulation in HFSS. (**a**) Frequency versus S(1,1) for different dielectric constant of the sensor substrate with *L* = 40 mm. (**b**) The extracted resonant frequency of the sensor with *L* = 40 mm, 100 mm, 200 mm, and 300 mm, respectively.

When the temperature increases from 25 to 1050 °C, the dielectric constant of the alumina substrate increases from 9.7 to 11.4 [16]. Here, the dielectric constant of the 99% alumina we used at 25 °C was 9.4 according to the data sheet provided by the producer. Hence, we performed a simulation in HFSS to probe the relationship between the temperature and the dielectric constant. Figure 4a shows the simulated frequency versus S(1,1) of the interrogation antenna under substrate dielectric constants from 9.4 to 11.8 with a sensor length *L* = 40 mm. With the increase in dielectric constant, the resonant frequency of the sensor decreased from 2.27 to 2.05 GHz. The amplitude of the S(1,1) tends to increase first and then decrease until the dielectric constant is 11.8. The extracted resonant frequencies under different dielectric constants where *L* = 40 mm, 100 mm, 200 mm, and 300 mm are plotted in Figure 4b, presenting good linearity with nearly equivalent slopes. Hence, it can be concluded that the patch length has little impact on the sensitivity of the sensor.

## 3. Measurement of the Sensor

**Figure 5:**
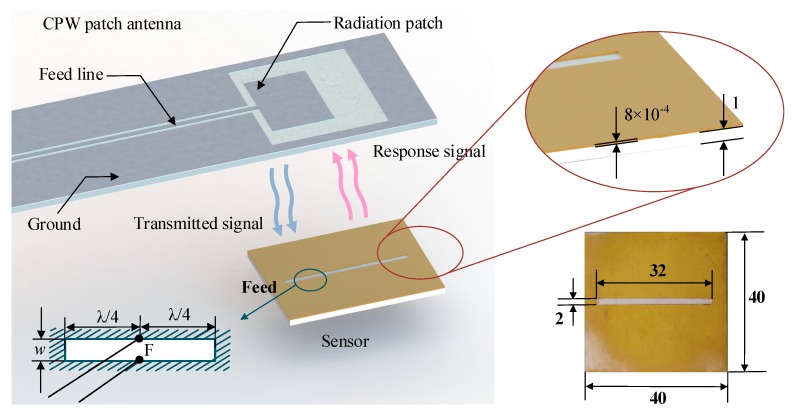
The electromagnetic principle of the sensor measurement.

For measurement, the fabricated sensor and the electromagnetic coupling principle between the readout antenna and the sensor is shown in Figure 5. A CPW patch antenna with a center frequency of 2.02 GHz, a relative frequency bandwidth of 49.5%, and a maximum vertical gain of 1.7 dB was used as the interrogation antenna to wirelessly interrogate the sensor with a sensing distance of 5 mm. The specific dimensions of the sensor are illustrated.

**Figure 6:**
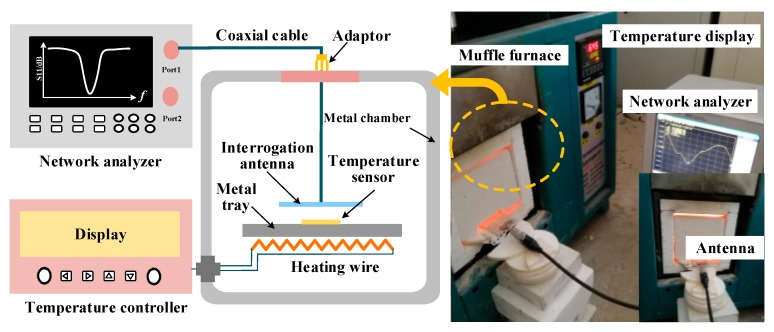
Measurement system of the temperature sensor.

The fabricated sensor was measured in a high-temperature muffle furnace using an off-the-shelf CPW patch antenna connected with an Agilent vector network analyzer (VNA) E5061B via a coaxial cable. The measurement system of the temperature sensor is shown in Figure 6. First, a series of sweep frequencies that contain the resonant frequency of the sensor were transmitted by the CPW interrogation antenna. We could see that the fabricated sensor had an initial resonant frequency of 2.31 GHz. A heating rate of 10 °C/min was then set for muffle furnace via the temperature controller and held for 5 min. For every 50 °C, a group of data were recorded by the VNA until the temperature had increased up to 800 °C.

**Figure 7:**
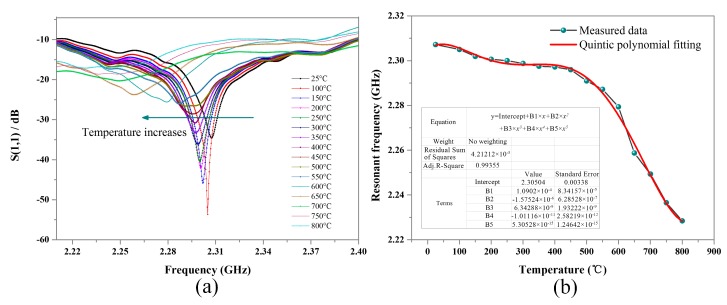
Measurement results for the temperature sensor. (**a**) The measured curves for frequency versus S(1,1). (**b**) The measured temperature versus extracted resonant frequency.

The measured curves for frequency versus S(1,1) are plotted in Figure 7a. The resonant frequency of the sensor decreases with the increase in the temperature in the range of 25–800 °C. When it was 25 °C, the signal of the sensor was easily detected due to the distinct negative peak laying around 2.31 GHz with S(1,1) of −35 dB. As the temperature increased, the value of S(1,1) first tended to increase and then decrease until the signal was difficult to be detected (at 800 °C). With the increase in temperature, the loss tangent of the alumina ceramic decreased first and then increased, so the dissipative energy of the sensor presented a similar variation. Figure 7b shows the measured temperature versus the extracted resonant frequency. The relationship between the measured temperature and the resonant frequency is not linear, thus presenting an irregular variation. For practical applications, we can obtain the measured temperature by looking up the relationship between temperature and resonant frequency gained in advance. Here, the plotted relationship between the temperature and the resonant frequency in the 25–800 °C range can be fitted as a quintic polynomial

(8) y=2.30504+1.0902e−4∗x−1.57524e−6∗x^2+6.34288e−9∗x^3−1.01116e−11∗x^4+5.30528e−15∗x^5.

The wireless temperature sensor we propose here has a low profile, high sensitivity, and a wide temperature sensing range. Table 2 shows visualized parameters of the RFID temperature sensor we fabricated and other kinds of wireless temperature sensors.

As we can see from Table 2, the slot radiation patch we designed has a simpler structure compared with conventional wireless SAW temperature sensors in [17]. Thin-film structures are required for a single-sided patch. It generally works at a microwave frequency band at the GHz level, while the LC sensor works at a lower frequency band in the 30–300 MHz range. Moreover, the thin-film structure is more sensitive than the LC sensor for its higher working frequency band. As for the temperature sensing range, compared with the LTCC-based sensor proposed in [8,10,17], the slot radiation patch would be able to operate at higher temperatures (above 1500 °C) if it were fabricated with Pt. For sensing distance, here the sensor was wirelessly measured with a distance of 5 mm in order to obtain a much larger |S(1,1)| and make the sensor tolerant to higher temperatures. However, for a Q factor higher than that of LC-based sensors, longer sensing distances are required and must be explored in further research. 

## 4. Discussion

A wireless and passive temperature sensor based on a microwave slot radiation patch was demonstrated. First, the sensor model was analyzed via Ansoft HFSS software for design of the dimensions on the principle of microwave backscatter based slot radiation. Then, the sensor was fabricated with a rectangular sheet of 99% alumina ceramic with a dimension of 40 mm × 40 mm × 1 mm prepared in micromechanics, with Au metallization in magnetron sputtering technology. The ready-made sensor was tested in a high-temperature furnace using an off-the-shelf microstrip antenna connected with a VNA via a coaxial cable. We can see from the measured results that the resonant frequency of the sensor decreases with the increase in temperature in the 25–800 °C range. This is due to the increase in the relative dielectric constant of the alumina substrate. The average sensitivity of the sensor is 101.94 kHz/°C. However, the linearity of the relationship between the measured resonant frequency and the temperature is not so good, which may be caused by various reasons, such as the temperature-dependent loss tangent of alumina and the mismatched coefficients of thermal expansion (CET) between alumina and Au. Our further research will focus on the improvement of the sensor linearity and the sensing distance. Moreover, the fabrication of a 3D printed sensor will be attempted, and this sensor, if the attempt is successful, will be integrated with an object of interest for in situ temperature measurement.

## Figures and Tables

**Table 1:** Properties of the 99% alumina ceramic.

Density [g/cm^3^]	Flexure Strength [MPa]	Young’s Modulus [GPa]	Dielectric Constant	Poisson’s Ratio
3.9	450	390	9.4	0.22

**Table 2:** Parameters of different temperature sensors.

Sensor Type	Profile	Sensitivity	Temperature Sensing Range	Sensing Distance	Working Frequency
Slot radiation patch sensor	40 mm × 40 mm × 1 mm	101.94 kHz/°C	25–800 °C	Maximum 14 mm	Around 2.31 GHz or higher
Resonator based microwave sensor in [10]	22 mm × 22 mm × 1.5 mm	0.24 MHz/°C	50–400 °C	30 mm	Around 2.28 GHz
LC resonance sensor in [8]	36 mm × 36 mm × 0.68 mm	Maximum 16.67 KHz/°C	25–700 °C	10 mm	Around 33 MHz
SAW based sensor in [17]	20 mm (dia)	/	Maximum +250 °C	Above 10 cm	Around 2.44 GHz
